# CD8 T Cell Hyperfunction and Reduced Tumour Control in Murine Models of Advanced Liver Disease

**DOI:** 10.1002/eji.70026

**Published:** 2025-08-04

**Authors:** Jood Madani, Jiafeng Li, Ma. Enrica Angela Ching, Agatha Vranjkovic, Katrina Jorritsma, Mohamed S. Hasim, Manijeh Daneshmand, Natasha Campeau, David A. Lawton, Salman Bagheri, Angela C. Cheung, Erin E. Mulvihill, Jennifer E. Bruin, Michele Ardolino, Angela M. Crawley

**Affiliations:** ^1^ Department of Biochemistry, Microbiology and Immunology University of Ottawa Ottawa Canada; ^2^ Inflammation and Chronic Disease Program Ottawa Hospital Research Institute Ottawa Canada; ^3^ Centre For Infection, Immunity and Inflammation University of Ottawa Ottawa Canada; ^4^ Department of Biology and Institute of Biochemistry Carleton University Ottawa Canada; ^5^ Cancer Research Program Ottawa Hospital Research Institute Ottawa Canada; ^6^ Nour Histopathology Consultation Services Ottawa Canada; ^7^ Division of Gastroenterology The Ottawa Hospital Ottawa Canada; ^8^ University of Ottawa Heart Institute Ottawa Canada

**Keywords:** CD8 T cells, immunotherapy, liver fibrosis, steatosis, tumour response

## Abstract

Immune dysfunction in liver disease contributes to significant morbidities, depending on liver damage severity and aetiology. We previously reported long‐lasting generalized CD8 T cell hyperfunction in chronic HCV infection with advanced fibrosis, yet its separation from viral and fibrosis‐driven effects, as well as clinical outcomes of advanced fibrosis, remains unclear. In a murine model of carbon tetrachloride‐induced progressive liver fibrosis, advanced fibrosis was observed by 12 weeks, with pathologies similar to those of human chronic HCV infection. Blood‐circulating CD8 T cells showed IFN‐γ and granzyme B (GrB) hyperfunction in response to anti‐CD3/28 stimulation, as well as impaired responses to ectopic tumour challenge and anti‐PD‐1/CTLA‐4 immunotherapy. Hyperfunction and impaired tumour responses were retained despite liver insult cessation. In a 45% HFD model, which induced steatosis and minimal fibrosis, IFN‐γ and GrB hyperfunction was also observed in blood‐circulating CD8 T cells. This study highlights a prolonged systemic CD8 T cell dysfunction acquired during progressive liver disease, associated with impaired antitumour and immunotherapy responses. These mirror the bulk CD8 T cell dysfunction observed in advanced liver diseases in humans, suggesting that these models could be valuable for future mechanistic studies aimed at identifying targets to help improve clinical outcomes in chronic liver disease.

## Graphical abstract

Blood CD8 T cells in mice with advanced liver fibrosis exhibit IFN‐γ and granzyme B hyperfunction, and animal responses to ectopic tumour challenge and immunotherapy are impaired.

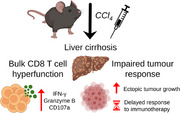

**Abbreviations**
ALT: alanine aminotransferase
AST: aspartate aminotransferase
CCl_4_
: carbon tetrachloride
DAA: direct‐acting antiviral
GrB: granzyme B
HCC: hepatocellular carcinoma
HCV: hepatitis C virus
HFD: high‐fat diet
IFN‐γ: interferon gamma
MASH: metabolic dysfunction‐associated steatohepatitis
MASLD: metabolic dysfunction‐associated steatotic liver disease
PBMC: peripheral blood mononuclear cell
SVR: sustained virologic response

## 1 Introduction

Around two million deaths annually are attributed to liver disease, representing 4% of all deaths globally [1]. An estimated 1.5 billion individuals, or up to 20% of the global population, are affected by chronic liver disease, caused predominantly by viral hepatitis, alcohol‐related liver disease, and increasing incidence rates of steatotic liver diseases [1, 2, 3]. The liver is a highly dynamic and important immunological organ, where resident and infiltrating immune cells are well‐positioned to respond to threats [4, 5]. Its extensive exposure to peripheral blood through its filtration role not only contributes to local responses but can result in a systemic phenotype reflective of organ pathology. Liver damage, including fibrosis, resulting from liver disease of many aetiologies, is consequently associated with major acquired defects in local and systemic immune functions [6, 7]. For example, chronic hepatitis C virus (HCV) infection disrupts innate and adaptive immune cell function, including the well‐characterized exhausted HCV‐specific CD8 T cells [8, 9, 10, 11, 12]. In addition to existing knowledge on antigen‐specific and liver‐resident immune cells, dysfunction of the bulk CD8 T cell population in the blood of HCV patients is increasingly recognized [13, 14, 15, 16, 17]. Our previous studies demonstrated increased blood CD8 T cell function, which we refer to as ‘hyperfunction’, in chronic HCV‐infected individuals with advanced liver fibrosis compared with minimal fibrosis, which persisted long after attaining sustained virologic response (SVR) with direct‐acting antiviral (DAA) therapy [17]. While T cell dysfunction is almost universally considered a loss of function (e.g., exhaustion of antigen‐specific anti‐viral or antitumor cells in chronic infections or cancer), observations of increased effector functions (e.g., interferon (IFN)‐γ and perforin) in bulk circulating CD8 T cells do not correlate with stronger antigen‐specific responses. On the contrary, CD8 T cell hyperfunction is associated with a reduced ability to fight cancer and infections [16, 18, 19]. Therefore, to ensure clarity, the term ‘hyperfunction’ will be used instead of ‘dysfunction’ when referring to elevated activity in bulk CD8 T cells. Metabolic‐dysfunction‐associated steatotic liver disease (MASLD) and its progressive pathology in metabolic dysfunction‐associated steatohepatitis (MASH) are also associated with increasing reports of immune cell dysfunction [20].

The underlying causes of many liver fibrosis aetiologies can be eliminated with various methods of intervention, allowing for hepatic tissue regeneration [21]. However, despite the spectacular results of DAAs and promising therapy avenues for MASLD, whether immune dysfunctions are reversible remains unclear. There are contrasting reports in the HCV literature, with observations of restoring exhausted HCV‐specific T cells [13, 14, 15, 16, 22, 23], and more recent epigenetic evidence of retained HCV‐specific T cell exhaustion, although fibrosis severity was not specified [24, 25, 26]. Longitudinal studies on immune dysfunction post‐fibrosis regression in other viral hepatitis aetiologies (e.g., chronic hepatitis B virus infection) and MASLD are also sparse.

Much has been learned about liver fibrosis progression in mouse models [27, 28]. The induction of liver fibrosis in rodents by exposure to the hepatotoxin carbon tetrachloride (CCl_4_) is a well‐established and highly reproducible model of liver fibrosis progression within 12 weeks [27, 28]. Pathology induced by CCl_4_ is characterized by fibrosis progression and periportal and portal‐to‐central fibrosis, both hallmark features of HCV infection pathology, and is reversible with insult removal [27, 28]. High‐fat ad libitum diets (HFD) resemble the metabolic features of human MASLD and MASH [29, 30] and develop significant liver steatosis and minimal fibrosis within 10–12 weeks [31].

To evaluate the potential of mouse models of liver fibrosis for translational studies of CD8 T cell hyperfunction in liver disease, we assessed organ pathology and CD8 T cell function in two models of chronic liver damage. Extensive characterization revealed a clear association between CD8 hyperfunction and pathological indicators of advanced liver damage, consistent with human observations in HCV infection and MASH (Vranjkovic; Li; Crawley, unpublished). To further demonstrate clinical relevance, the models were challenged with an experimental ectopic tumour known to be controlled by effective CD8 T cell responses, and liver disease animals showed impaired tumour control. As a complement to this, the potential compromise of immune responses in chronic liver disease was assessed by treating tumour‐bearing animals with anti‐PD‐1/CTLA‐4 immunotherapy. These findings highlight shared mechanisms of CD8 T cell dysfunction across various liver disease aetiologies and support the use of these models for future studies aiming to identify and target underlying mechanisms.

## 2 Results

### 2.1 CCl_4_‐Treated Mice Develop Advanced Fibrosis Similar to Pathologies Observed in Human Chronic HCV

**FIGURE 1:**
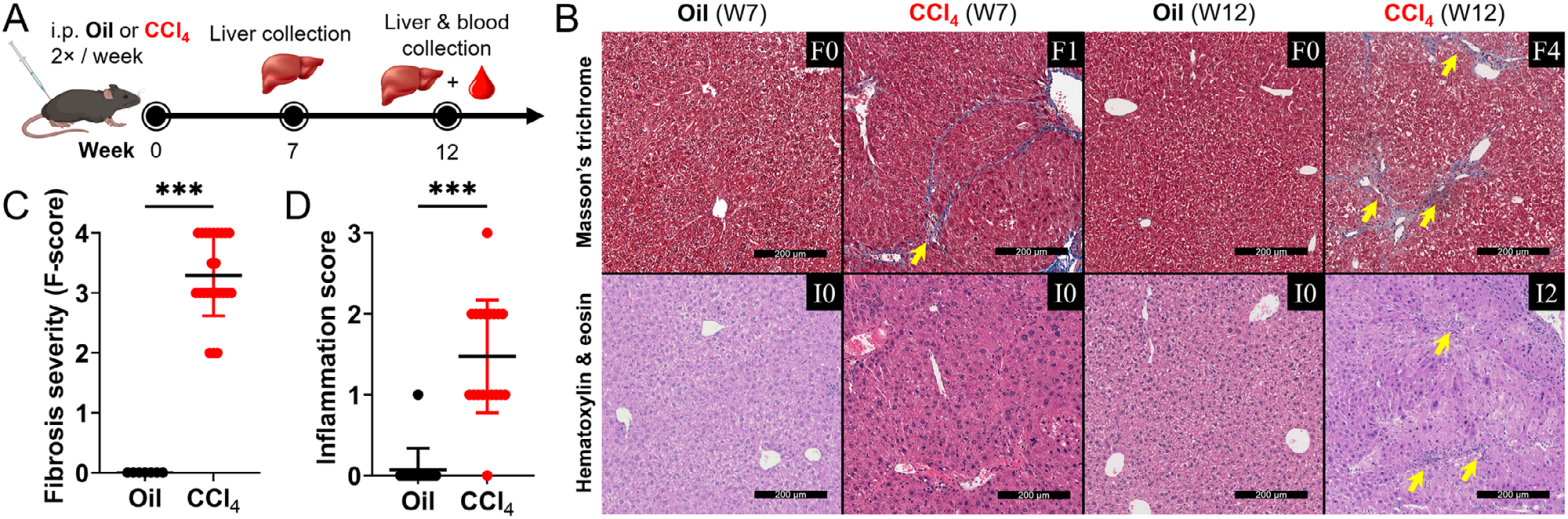
**Advanced liver fibrosis is induced in C57BL/6 mice by prolonged CCl_4_ exposure. (A)** Mice were administered CCl_4_ or olive oil (vehicle control) for ≈12 weeks, and liver tissue and blood were collected to assess liver disease severity and CD8 T cell function. **(B)** Representative Masson's trichrome (top) and hematoxylin & eosin (bottom) staining of mouse liver tissues at 7 and 12 weeks into CCl_4_ exposure, where progressive liver fibrosis and inflammation development was observed and respective pathologist‐determined scores are indicated. Yellow arrows highlight areas of fibrosis (top) or lobular inflammation (bottom). Scale bars: 200 µm. Summary of **(C)** liver fibrosis and **(D)** inflammation severity induced by ≈12 weeks of CCl_4_ exposure across three independent experiments are shown. Fibrosis scores were determined using the Metavir scoring system (F0 no fibrosis, F0‐1 minimal, F2‐3 intermediate, F3‐4 advanced fibrosis, F4 cirrhosis) while inflammation was scored across stages (I0‐I3). Study group sizes may range from 3–10, depending on the experiment. Comparisons by unpaired Student's *t*‐test, *p ≤ 0.05, **p ≤ 0.01.

To examine the effect of advanced liver fibrosis progression on CD8 T cell function without viral infection as a confounding factor, the CCl_4_ hepatotoxin‐based mouse model of chronic liver fibrosis was adapted from established protocols (27, 32). Intraperitoneal (i.p.) administration of 1.0 mL/kg of CCl_4_ twice a week for 12 weeks (Figure 1A) was well tolerated in C57BL/6 mice, and weight gain over time was no different from that of control mice (Figure S1A).

Liver damage progression was assessed by fibrosis severity scoring following the Metavir system [33, 34]. After 7 weeks of CCl_4_ injections, moderate peripheral and bridging fibrosis, scoring ≈F1 (Figure 1B), was observed, compared with the healthy tissue profile of oil vehicle controls (scoring ≈F0). Oil‐treated mice also displayed little to no signs of liver damage compared with untreated animals (Figure S1B). By ≈12 weeks of CCl_4_ exposure, tissue damage progressed to severe diffuse fibrosis (scoring ≈F3‐4, Figure 1B,C) with focal necrosis and surrounding mixed moderate inflammation (Figure 1B,D). Impacted liver function was confirmed by observations of increased plasma levels of aspartate aminotransferase (AST) and alanine aminotransferase (ALT) in CCl_4_‐treated mice at the peak of fibrosis (i.e., ≈12 weeks) compared with control mice (Figure S1C), as expected and observed by others [35, 36]. The plasma AST/ALT ratio (1.737 ± 0.352, mean ± SD) observed in CCl_4_‐treated mice reflects the typical ratio of ≈2 observed clinically during fibrogenic chronic liver damage [37]. In both male and female mice, the fibrosis pathology resembled that of chronic HCV infection in humans, with characteristic bridging fibrosis leading to cirrhosis [28].

These results highlight the validity of CCl_4_ exposure in modelling progressive liver fibrosis, reflecting the pathology of chronic HCV with fibrosis progression, confirming it as a suitable model to investigate immune responses in advanced liver disease uncoupled from the effects of viral infection.

### 2.2 CD8 T Cells Exhibit Hyperfunction in Mice with CCl_4_‐Induced Advanced Fibrosis

**FIGURE 2:**
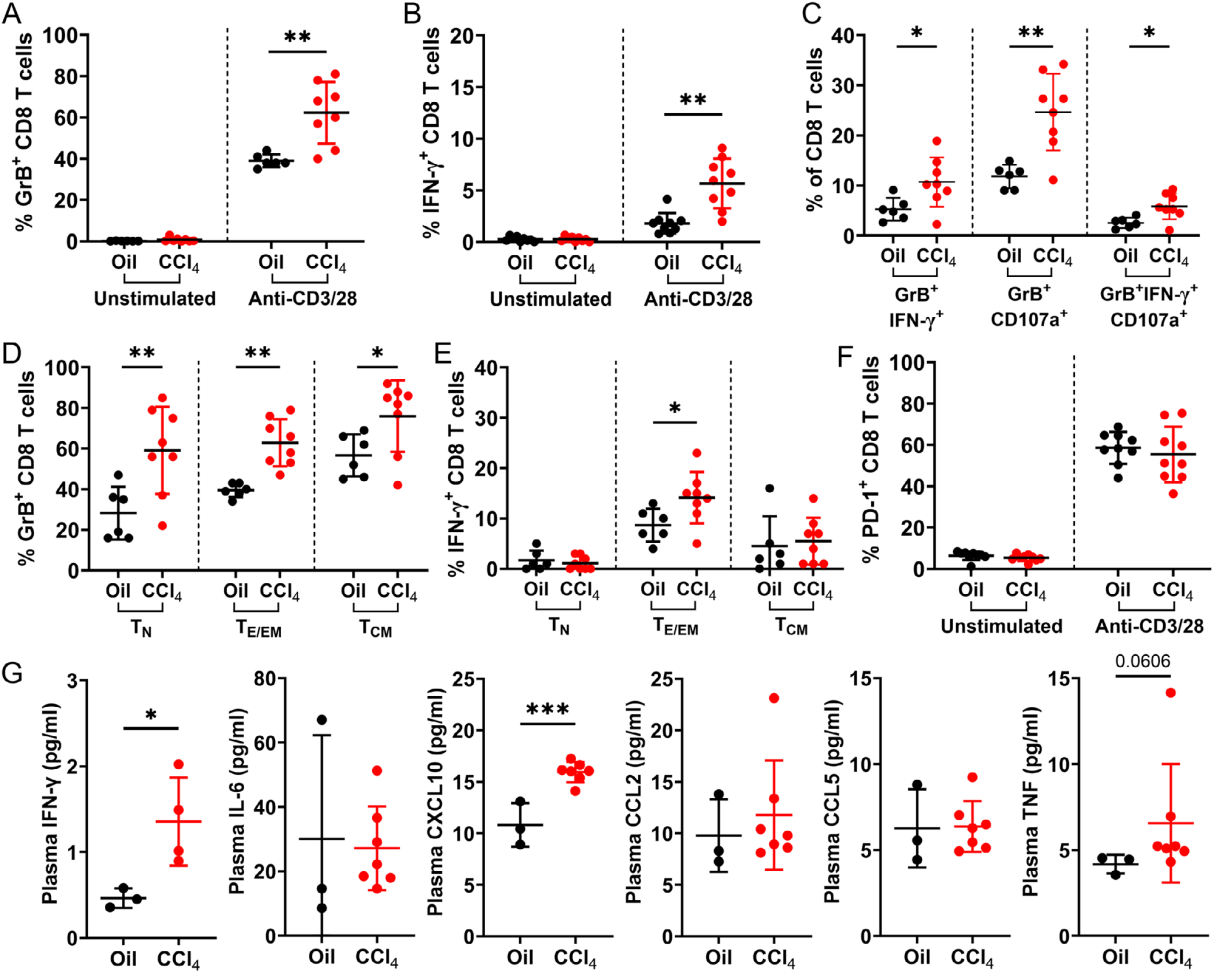
**Mice with CCl_4_‐induced advanced liver fibrosis exhibit CD8 T cell hyperfunction**. CD8 T cell function was assessed after anti‐CD3/28 stimulation of PBMCs from mice with advanced liver fibrosis induced by ≈12 weeks of CCl_4_ exposure, compared with oil control mice. Data from several independent experiments are shown in the figure panels. Hyperfunction in bulk CD8 T cells was observed for both **(A)** GrB and **(B)** IFN‐γ expression. The remaining figure panels display results from CD8 T cells stimulated with anti‐CD3/CD‐28 antibodies, comparing Oil controls to CCl_4_‐treated mice: **(C)** Increased proportions of poly‐functional (GrB^+^IFN‐γ^+^, GrB^+^CD107a^+^, GrB^+^IFN‐γ^+^CD107a^+^) CD8 T cells were also observed. This hyperfunction was seen in **(D)** T_N_, T_E/EM_, and T_CM_ subsets for GrB expression, and **(E)** T_E/EM_ subset for IFN‐γ expression. **(F)** The expression of PD‐1 on CD8 T cells remained unchanged. **(G)** Increased plasma levels of inflammatory cytokines were observed in mice with CCl_4_‐induced advanced fibrosis. Comparisons by unpaired Student's *t*‐test, *p ≤ 0.05, **p ≤ 0.01, ***p ≤ 0.001.

The function of CD8 T cells from peripheral blood mononuclear cells (PBMCs) in CCl_4_‐treated mice was evaluated at peak liver fibrosis, by *ex vivo* stimulation with anti‐CD3 and anti‐CD28 antibodies for 48 h. CD8 T cell function was evaluated by flow cytometry following the gating strategy outlined in Figure S2. The proportion of stimulated CD8 T cells expressing cytotoxic granzyme B (GrB) in CCl_4_‐treated mice was significantly higher than in control mice (Figure 2A). Similarly, the proportion of CD8 T cells expressing immune‐modulating IFN‐γ was also significantly elevated in mice exposed to CCl_4_ compared with controls (Figure 2B). The proportions of bi‐functional cells, such as GrB^+^IFN‐γ^+^ and GrB^+^CD107a^+^ cells, were also increased (Figure 2C), as were the proportions of poly‐functional GrB^+^IFN‐γ^+^CD107a^+^ CD8 T cells (Figure 2C).

In our previous report on human HCV infection, generalized CD8 T cell hyperfunction was particularly prominent in naïve (T_N_) and effector memory (T_EM_) cells (17). To assess whether similar subsets also drive CD8 T cell hyperfunction in the CCl_4_ model, CD8 T cell function was assessed in CCl_4_‐treated mice across T_N_ (CD44^−^CD62L^+^), effector and effector memory (T_E/EM_, CD44^+^CD62L^−^), as well as central memory (T_CM_, CD44^+^CD62L^+^) subsets. In stimulated CD8 T cells from mice treated with CCl_4_, increased proportions of GrB^+^ cells were observed across all cell subsets compared with the production of this functional protein by anti‐CD3/CD‐28‐stimulated naïve CD8 T cells in control mice (Figure 2D). Increased proportions of IFN‐γ^+^ CD8 T cells were also detected in the T_E/EM_ subsets (Figure 2E). The upregulation of PD‐1 by stimulated CD8 T cells in mice treated with CCl_4_ was similar to that of controls (Figure 2F), suggesting a consistent activation and subsequent exhaustion in response to stimulation.

Finally, inflammatory plasma cytokine levels, namely of IFN‐γ, CXCL10, and TNF, were increased in CCl_4_‐treated mice at peak liver fibrosis, compared with oil controls (Figure 2G). Taken together, we show here hyperfunction of blood circulating CD8 T cells in a mouse model of CCl_4_ hepatotoxin‐induced liver fibrosis, adapted for the context of advanced stages of liver fibrosis and uncoupled from viral hepatitis. This hyperfunction is marked by increased cytokine production and cytotoxic potential across multiple cell subsets.

### 2.3 Bulk CD8 T Cell Hyperfunction in Mice with CCl_4_‐Induced Advanced Fibrosis Persists After Regression

**FIGURE 3:**
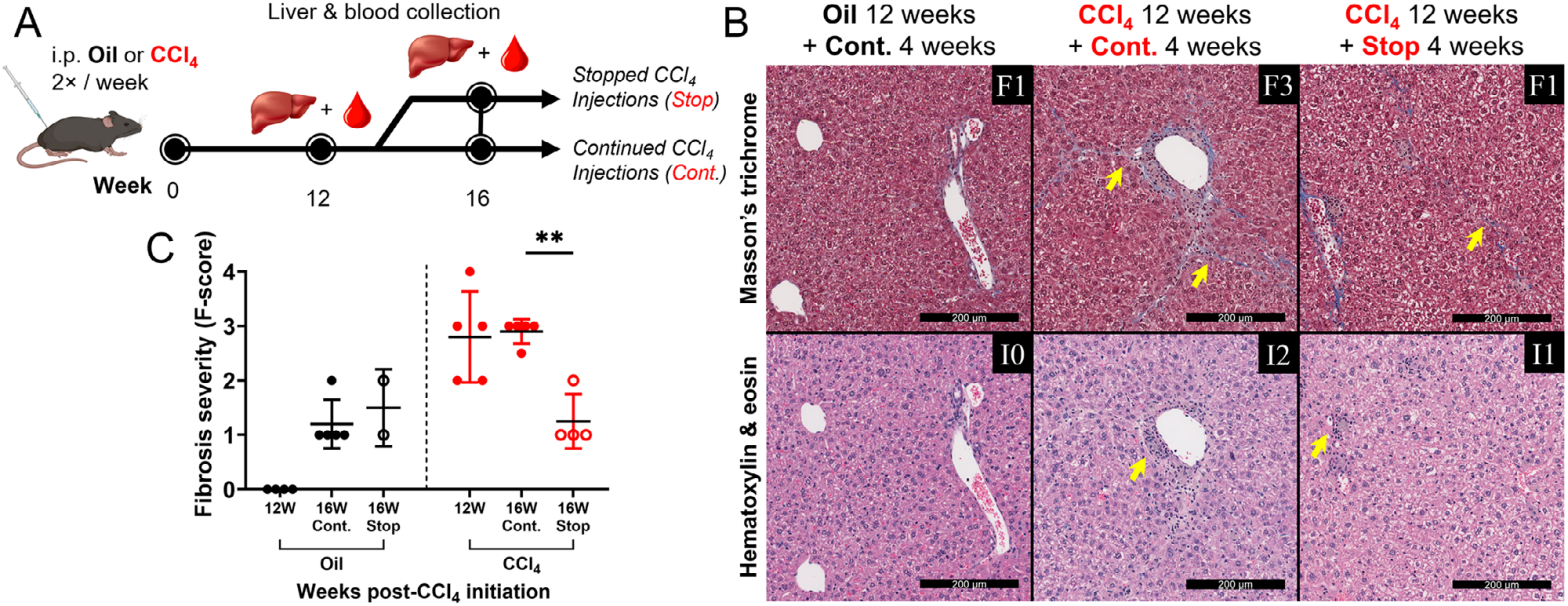
**Advanced liver fibrosis regresses after the cessation of CCl_4_ exposure. (A)** At peak CCl_4_‐induced liver fibrosis, mice underwent either (1) continued (Cont.) CCl_4_ administration, or (2) stopped (Stop) CCl_4_ treatment. **(B)** Representative Masson's trichrome (top) and hematoxylin & eosin (bottom) staining of mouse liver tissues after continued or ceased CCl_4_ treatment. Yellow arrows highlight areas of fibrosis. Scale bars: 200 µm. **(C)** Summary of liver fibrosis severity after 16 weeks of continuous CCl_4_ exposure, or after 12 weeks of CCl_4_ exposure followed by 4 weeks of exposure cessation (Metavir scoring: F0 no fibrosis, F0‐1 minimal, F2‐3 intermediate, F3‐4 advanced fibrosis, F4 cirrhosis). Multiple comparisons by 1‐way ANOVA with Dunnett's post‐test, **p ≤ 0.01.

In humans, the removal of chronic liver insult, such as achieving SVR (i.e., viral clearance) in HCV infection, can enable the regression of liver fibrosis, yet it depends on the initial liver damage severity and comorbidities [38, 39]. It has been reported that CCl_4_‐induced liver fibrosis in mice is reversed around 2–3 weeks post‐treatment cessation [40]. In our previous report on human HCV infection, CD8 T cell hyperfunction persisted 24 weeks post‐SVR in individuals whose advanced fibrosis had partially regressed [17]. Therefore, to assess CD8 T cell function following liver injury regression in the CCl_4_ model of advanced fibrosis, hepatotoxin administration was stopped in a randomized subgroup of mice after 12 weeks, and liver pathology was assessed 4 weeks later (Figure 3A). By 4 weeks post‐CCl_4_‐cessation, liver fibrosis severity reversed significantly from F3–F4 to F1–F2, while fibrosis persisted with continued CCl_4_ exposure (Figure 3B,C).

**FIGURE 4:**
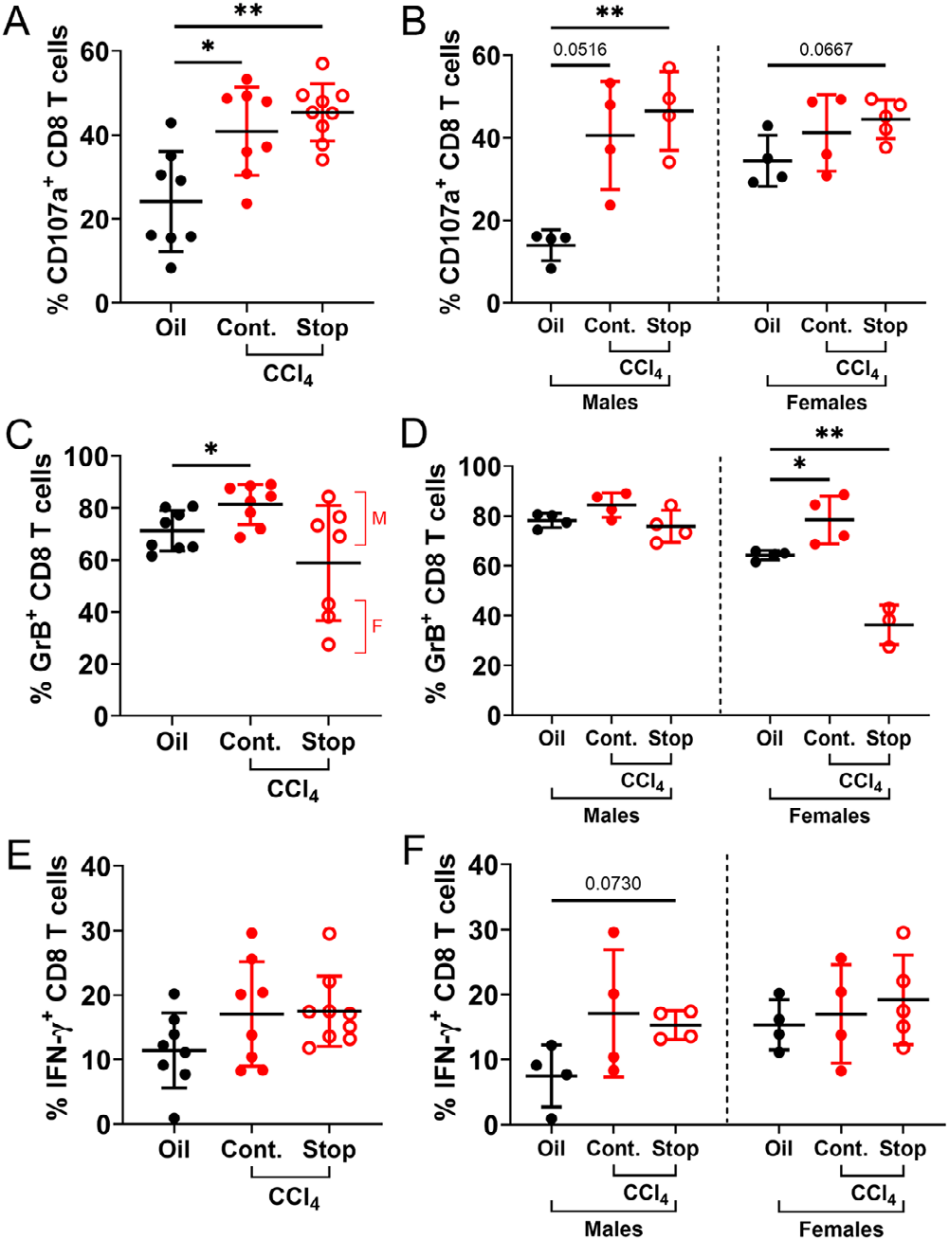
**Bulk CD8 T cell hyperfunction is not fully reversed despite regression of advanced liver fibrosis**. CD8 T cell function was assessed in mice after 12 weeks of CCl_4_ exposure, followed by 4 weeks of either CCl_4_ cessation or continuation. **(A)** The proportion of CD107a^+^ CD8 T cells is significantly higher in CCl_4_‐treated mice with both continued or stopped exposure, with **(B)** hyperfunction more prominently observed in male mice. By contrast, **(C)** GrB hyperfunction in CD8 T cells was not sustained after CCl_4_ cessation. M: Males, F: Females. **(D)** Both GrB hyperfunction and functional regression were more prominently observed in female mice compared with male mice. **(E)** IFN‐γ expression, while not statistically significant between groups, followed a similar increased and persistent function pattern as CD107a, including when stratified by sex **(panel F)**. Multiple comparisons by 1‐way ANOVA with Dunnett's post‐test, *p ≤ 0.05, **p ≤ 0.01.

We next assessed whether the CD8 T cell hyperfunction observed at peak liver fibrosis (≈12 weeks CCl_4_) persisted after liver fibrosis regression. Bulk CD8 T cell expression of CD107a, GrB, and IFN‐γ are significantly elevated in mice with continued CCl_4_ exposure compared with oil controls in this and previous animal study groups. These three functional output levels were sustained in mice where liver fibrosis severity was regressed by 4 weeks of CCl_4_ treatment cessation in males and females (Figure 4). A noted exception to the results of a combined male and female study shows that the three females reduced GrB expression following CCl_4_ cessation. The latter is in contrast to two independent female‐only studies where no differences were observed in the T cell functions between mice with continued CCl_4_ treatment compared with those whose treatment was stopped (data not shown). Therefore, these results suggest that the observed bulk CD8 T cell hyperfunction is overall retained despite the regression of advanced liver fibrosis following cessation of CCl_4_ exposure.

### 2.4 Responses to Tumour Growth and Checkpoint Inhibitors Are Impaired in CCl_4_‐induced Advanced Fibrosis

**FIGURE 5:**
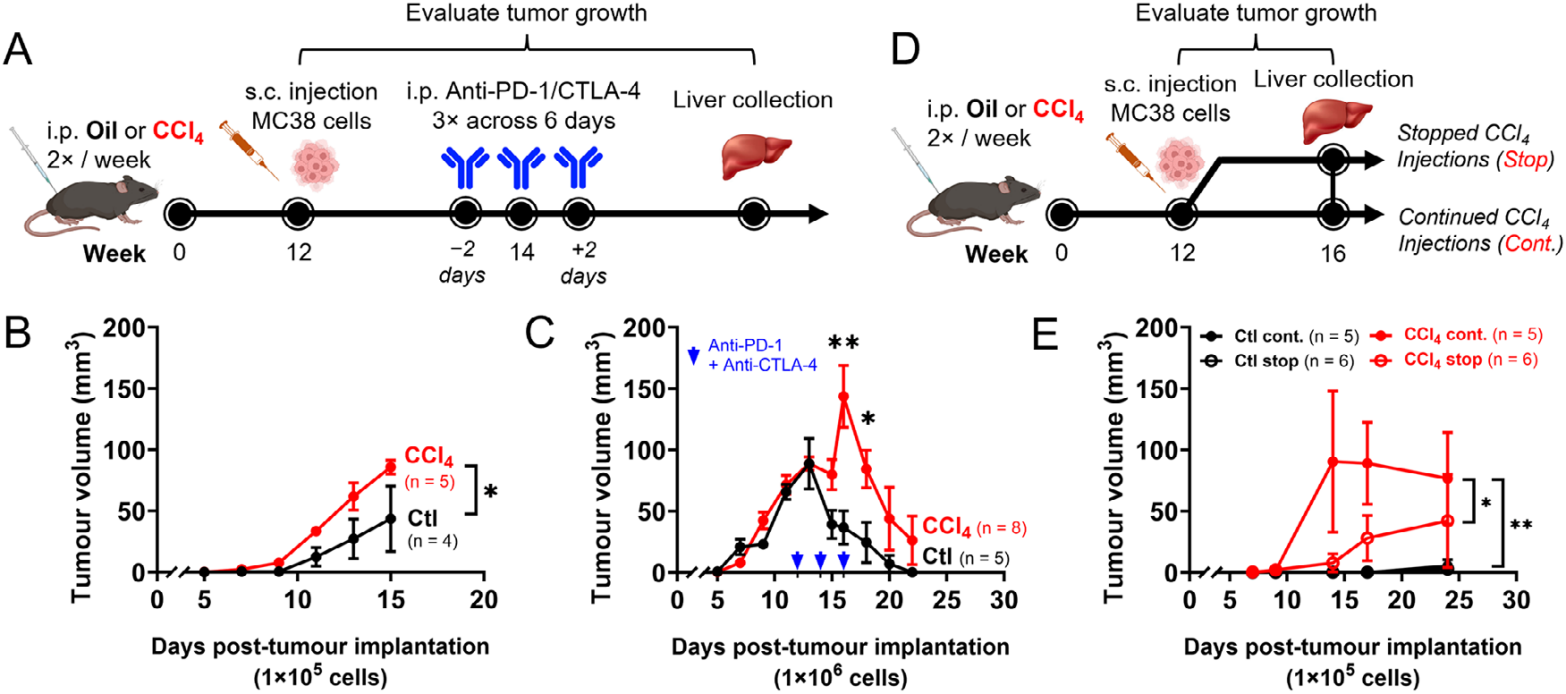
**Ectopic tumour growth is exacerbated and response to checkpoint inhibitor therapy is delayed in mice with CCl4‐induced advanced fibrosis. (A)** Mice with CCl_4_‐induced advanced fibrosis were administered MC38 tumour cells, followed by anti‐PD‐1 and anti‐CTLA‐4 immunotherapy after tumour growth. **(B)** A low dose of MC38 cells (1×10^5^) resulted in CCl_4_‐treated mice responding significantly poorer to tumour growth compared with oil controls. **(C)** Higher doses of MC38 cells (1 × 10^6^) lead to comparable tumour growth in CCl_4_‐treated and control mice, where CCl_4_‐treated mice exhibited delayed and weakened responses to tumour growth under anti‐PD‐1 and anti‐CTLA‐4 immunotherapy. **(D)** At peak liver fibrosis, mice were put on continued or ceased CCl_4_ treatment for an additional 4 weeks, prior to MC38 tumour challenge (1 × 10^5^ cells). **(E)** Impaired tumour responses observed in CCl_4_‐treated mice were not fully reversed after fibrosis regression. Multiple comparisons by 2‐way ANOVA with Šídák's post‐test, *p≤0.05, **p≤0.01.

Next, clinically relevant immunological consequences of CD8 T cell hyperfunction in chronic advanced liver fibrosis were assessed. At peak liver fibrosis, where CD8 T cell hyperfunction was observed, mice were challenged with subcutaneous (s.c.) implantation of MC38 colon carcinoma cells, followed by anti‐PD‐1 and anti‐CTLA‐4 combined immunotherapy after tumour volumes reached ≈75 mm^3^ (Figure 5A). Challenge by MC38 cells is a well‐established transplantable tumour model where clearance is heavily mediated by CD8 T cells [41, 42].

Without immunotherapy, a low dose of implanted MC38 cells (1 × 10^5^) resulted in significantly increased tumour growth over time in mice exposed to CCl_4_ compared with controls (Figure 5B). Under an increased tumour burden (1 × 10^6^ implanted cells), where parallel tumour growth was induced in both groups, mice with CCl_4_‐induced advanced liver fibrosis showed both a delayed and weakened anti‐tumour response upon treatment with anti‐PD‐1 and anti‐CTLA‐4 immunotherapy (Figure 5C).

Given that bulk CD8 T cell hyperfunction is not fully restored with the removal of liver insult and concurrent organ regeneration, the impact of sustained CD8 T cell hyperfunction on anti‐tumour responses was investigated. At peak fibrosis with detectable bulk CD8 T cell hyperfunction, mice in a randomized subgroup underwent CCl_4_ treatment cessation for 4 weeks, leading to fibrosis regression, after which all mice were challenged with 1 × 10^5^ implanted MC38 cells (Figure 5D). With continued CCl_4_ administration, nearly all mice (4/5) rapidly (≈14 days posttumour cell implantation) developed tumours that were significantly larger by ≈25 days posttumour cell implantation, compared with mice that underwent CCl_4_ cessation (Figure 5E). Removal of liver insult resulted in mixed tumour control, with 3/6 mice permitting slow tumour growth while the remaining mice managed to control tumour growth throughout the study (Figure 5E). This mixed tumour control with CCl_4_ cessation was not statistically different over time compared with the oil control group, which, in this study group, consistently prevented tumour development (Figure 5E).

These results show that bulk CD8 T cell hyperfunction in mice with CCl_4_‐induced advanced liver disease associates with impaired anti‐tumour responses, weakened and delayed responses to anti‐PD‐1 and anti‐CTLA‐4 immunotherapy, and that this dysfunction may persist after fibrosis regression.

### 2.5 Bulk CD8 T Cell Hyperfunction Also Associates with Steatosis and Ballooning with Minimal Fibrosis

**FIGURE 6:**
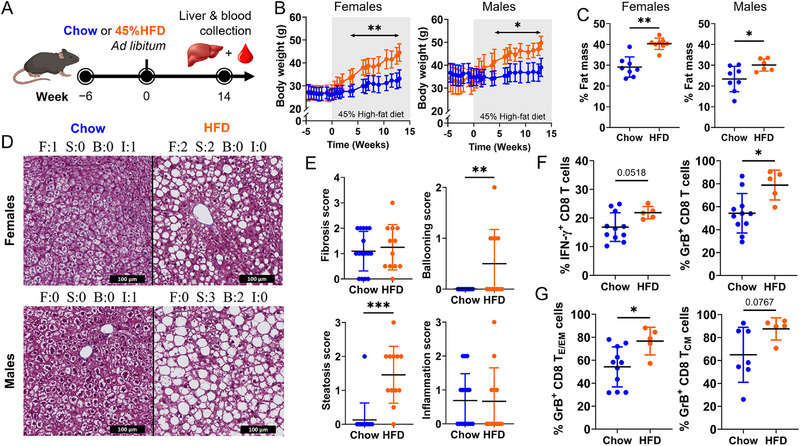
**High‐fat diet induces CD8 T cell hyperfunction associated with liver steatosis and ballooning in the absence of significant fibrosis. (A)** C57BL/6 mice were placed on a 45% HFD for 14 weeks prior to blood collection and liver collection. **(B)** Both female (left) and male (right) mice under HFD significantly gained weight compared with chow‐fed controls. **(C)** Both female (left) and male (right) mice significantly gained percent fat mass when assessed at ≈11 weeks of HFD compared with chow‐fed mice. **(D)** Mice fed HFD for 14 weeks developed steatotic liver disease with fibrosis (F), steatosis (S), ballooning (B), and inflammation (I). **(E)** Summarized steatotic liver disease scores across all mice, with significantly increased steatosis and ballooning induced by HFD. **(F)** Bulk CD8 T cell hyperfunction in HFD‐fed mice compared with controls, as observed by increased IFN‐γ (left) and GrB (right) expression. **(G)** GrB hyperfunction was predominantly observed in T_E/EM_ (left) and T_CM_ (right) subsets in mice fed HFD. Body weight comparisons by two‐way ANOVA with Šídák's post‐test; fat mass, disease scores, and T cell function comparisons by unpaired Student's *t*‐test; *p ≤ 0.05, **p ≤ 0.01, ***p ≤ 0.001.

Finally, we aimed to determine whether systemic CD8 T cell hyperfunction is a generalized feature of advanced liver disease pathologies beyond fibrosis. Preliminary findings in humans showed bulk CD8 T cell hyperfunction in MASLD with advanced fibrosis compared with healthy individuals (Figure S3). C57BL/6 mice were fed a 14‐week HFD regimen consisting of 45% energy from fat, followed by blood and liver tissue collection to assess CD8 T cell function and liver disease severity score, respectively (Figure 6A). Both male and female mice fed HFD significantly gained body weight and percent fat mass compared with control mice on a regular chow diet (Figure 6B,C). After 13 weeks, HFD‐fed mice were also hyperglycaemic (Figure S4A,B) and hyperinsulinemic (Figure S4C,D) compared with control mice, both hallmarks of type II diabetes in humans, including in MASLD [43, 44, 45, 46].

Histopathological scoring of liver disease severity revealed low to moderate steatosis and hepatocyte ballooning in mice fed HFD, significantly differing from controls (Figure 6D,E). While HFD‐induced liver injury appears to be prominently driven by steatosis in female mice as opposed to hepatocyte ballooning in male mice, the combined non‐alcoholic fatty liver disease Activity Score was significantly increased in both sexes compared with chow‐fed mice (Figure S5A, top vs. bottom). Interestingly, in chow‐fed mice, we observed minimal fibrosis scoring between F0 and F2 at endpoint, accompanied by low levels of inflammation (Figure 6D,E), which was also present in HFD‐fed mice. Liver AST and ALT levels were increased in the plasma of HFD‐fed mice at endpoint compared with controls (Figure S5B), as expected in mice HFD‐induced liver damage [49], with an AST/ALT ratio of 7.175 ± 8.677 (mean ± SD) in males and 5.733 ± 3.108 in females. In PBMCs from HFD‐fed mice stimulated with anti‐CD3 and anti‐CD28 antibodies, a greater proportion of bulk CD8 T cells expressed IFN‐γ and GrB compared with controls (Figure 6F). In addition, the systemic hyperfunction in GrB was also observed in T_E/EM_ and T_CM_ subsets (Figure 6G).

These results indicate that in mice with diet‐induced steatotic liver disease with pathologies resembling human MASLD, blood‐circulating bulk CD8 T cells also exhibit hyperfunction. Taken together with observations of cellular hyperfunction in CCl_4_‐treated mice with advanced liver disease, this suggests that systemic CD8 T cell hyperfunction is not limited to advanced fibrosis and may occur across different aetiologies of chronic liver disease.

## 3 Discussion

Immune cell dysfunction is a hallmark feature of chronic liver disease, associated with impaired antiviral responses to hepatic infection and increased risk of hepatocellular carcinoma (HCC). However, systemic immune cell activation and hyperfunction, coupled with systemic inflammation in the context of advanced stages of liver damage, reported by us and others in CD8 T cells, is not well understood. This study found a consistent induction of advanced liver fibrosis pathology after 12 weeks of hepatotoxin injection (i.p.), by adapting a protocol from previous studies in rats [50]. These data show that persistent presentation of severe liver pathology was associated with increased inducible expression of IFN‐γ and GrB in circulating CD8 T cells, emulating previous observations in chronic HCV infection with cirrhosis [17]. Importantly, this hyperfunction was somewhat sustained following fibrosis regression post‐CCl_4_ cessation. This generalized dysfunction was also associated with poor control of ectopic tumour growth and a delayed response to immunotherapy. This not only confirmed the association of CD8 T cell hyperfunction with liver fibrosis severity, but also decoupled this from hepatic viral infection. These results also ascribe potential clinical relevance to long‐term outcomes and therapeutic responsiveness in the context of advanced liver fibrosis. A complement of similar cell dysfunction findings in a model of diet‐induced chronic liver disease, causing liver steatosis without significant fibrosis, expands the scope of impact of this phenomenon to different etiologies of chronic liver disease.

Much has been learned about the processes of liver fibrosis progression in mouse models of liver fibrosis [28]. While there is no single animal model that recapitulates all the features of liver disease, the CCl_4_ model was consistent in its periportal and portal‐to‐central fibrosis pathology and moderate inflammation, as in HCV infection [28]. The associated CD8 T cell hyperfunction resembled that observed in HCV‐infected individuals with cirrhosis, further confirming the suitability of this model to evaluate immune cell hyperfunction in advanced liver fibrosis. Thus, the model was well‐suited to decouple advanced liver fibrosis from virus, confirming our hypothesis that immune cell hyperfunction will occur in advanced liver disease of varying etiologies. Although there exist murine models permitting HCV infection, these are not fully immunocompetent, nor can they fully exhibit liver tissue pathologies reflective of human HCV [51, 52]. Consequently, the impact of fibrosis progression and severity on immune function remains relatively poorly understood.

The CCl_4_ hepatotoxin has previously been shown to enhance and then subsequently diminish mouse T cell responses to non‐specific mitogens after 7 and 23 days, respectively, while impairing T cell‐dependent antibody responses when administered i.p. [53, 54]. Another short‐term 2‐week course study of CCl_4_ treatment (i.p.) resulted in impaired responses to *Listeria monocytogenes* and *Streptococcus pneumoniae*, both infections in which CD8 T cell responses play an important role in clearance [55]. These latter studies focused on the acute effects of CCl_4_, when significant organ fibrosis is not yet established. Similarly, studies into the sex effects of advanced liver disease and its associated immunological consequences remain sparse. Our results, when stratified by sex where possible, appear to show overall worse liver disease outcomes in male compared with female mice, along with reduced capacity for immunological restoration. Estrogens have been shown to inhibit stellate cell activation and fibrogenesis [56], the effects of which can be observed in human MASLD, where reported fibrosis severity is higher in men and post‐menopause women, compared with pre‐menopause women [57]. To date, the model has not, to our knowledge, been evaluated in mice to study chronic advanced liver fibrosis and its long‐term immunological effects. This study permits the rationale for future studies exploring the potential underlying mechanisms, some of which we have recently uncovered in an RNA sequencing analysis of blood CD8 T cells in chronic HCV infection, showing lasting gene expression patterns post‐cure [58].

The notion of T cell hyperfunction in chronic disease may be associated in part with generalized immune activation. This is widely described in chronic viral infections, characterized by increased HLA‐DR and CD28 expression, as well as other biomarkers of inflammation such as serum lipopolysaccharide and soluble CD14 [59, 60]. A recent report has found CD8 T cell hyperfunction in a mouse model of MASH, associating it with reduced tumour surveillance and poor response to immunotherapy for HCC [61]. Moreover, in this model, prophylactic immunotherapy elevated CD8 T cell hyperfunction further and paradoxically increased HCC tumour burden. This sheds light on our overall hypothesis that local and systemic generalized CD8 T cell hyperfunction impairs immune surveillance, a central feature of CD8 T cell response, resulting in susceptibility to challenge. As it has been extensively shown that MC38 tumours face potent CD8 T cell responses [42], our data support the notion that liver fibrosis severely compromises the immune system to the point where it is incapable of responding to tumour challenge. Whether the accumulation of aged or dysregulated T cells in the fibrotic liver, thought to impact naïve T cell replenishment [62], is responsible for the observed CD8 T cell hyperfunction and impaired tumour surveillance remains to be determined in this model. T cell infiltration into the liver was observed (data not shown), but whether this is due to an impediment to T cell clearance remains to be determined, as markers of T cell age or apoptosis were not included in the study panel.

Although the removal of liver insult, such as therapeutic elimination of HCV infection (i.e., SVR), can halt liver disease progression, immune dysfunction may persist. Whether the T cell alterations are caused by liver disease and fibrosis or by the associated systemic inflammation that may trigger liver damage is a difficult question to disentangle in this model. In HCV infection, liver fibrosis regression can occur, depending on severity, following SVR with highly effective antiviral therapy. In cirrhosis, the occurrence of incremental regression (i.e., single F‐stage reduction), if at all, is approximately 50% [38, 63]. We have observed this in previous clinical studies of HCV^+^ individuals with cirrhosis 24 weeks post‐SVR with DAA therapy [64]. Nevertheless, we observed sustained CD8 T cell hyperfunction long after cure [17]. Whether a lack of liver fibrosis regression is reflected in recovery from immune cell dysfunction is not known, and host or pathogen contributions therein are unclear. In a rat model of chronic CCl_4_ exposure, there was progressive loss of fibrotic matrix 28 days following the cessation of toxin administration [28, 50].

We report here in mice that a 12‐week CCl_4_ protocol achieving maximal fibrosis can subsequently be fully reversed (Figure 3), yet CD8 T cell hyperfunction persisted at this measured time (Figure 4). While it is possible that a longer follow‐up may have shown stronger immune cell functional recovery, it remains likely that persistent dysfunction would have been observed, as we and others have described in bulk or HCV‐specific CD8 T cells long after DAA‐mediated viral clearance. In either case, the removal of liver insult or any degree of fibrosis regression does not readily restore CD8 T cell function over time. Similar retention of immune dysfunction has been reported in cirrhotic individuals when monitoring HCV‐specific CD8 T cell impairment post‐DAA cure [65, 66, 67]. Our recently reported gene expression profiling of hyperfunctional bulk CD8 T cells in HCV^+^ individuals post‐DAA also suggests several pathways that may underlie this dysfunction, including Hedgehog signalling and T cell activation‐related metabolic regulation [58]. Recent reports ascribing epigenetic imprinting of these cells, although the study subject fibrosis severity is not specified [24, 25, 26]. Our findings, and those of others, suggest inherent lasting trauma to the cells, whether T cell receptor driven from ongoing antigen exposure leading to impairment, or through other environmental signals that persist despite removal of liver insult, ultimately resulting in cellular hyperfunction. This invokes the potential for lasting epigenetic modifications previously reported in other models of chronicity [68] and is the subject of future research, which could lead to identifying potential underlying mechanisms or novel immunorestorative targets. Pivotal studies in the murine chronic LCMV model have revealed epigenetic modifications as an underlying feature of CD8 T cell dysfunction [69].

In addition, since any derangement of immune surveillance, an important aspect of CD8 T cell function, could contribute to cancer development, we also evaluated the impact on tumour challenge and immunotherapy in this model. To do so, we employed the MC38 cell line, a commonly used transplantable mouse tumour model [41] known to induce a potent CD8 T cell response, to investigate anticancer immunity [42]. Our data shows a lack of tumour control despite the resolution of liver tissue damage (Figure 5E). These findings offer translational relevance in associating generalized systemic CD8 T cell hyperfunction in advanced liver disease with impaired responses to tumour challenge. In compensated cirrhosis, there is immune cell activation and systemic inflammation, as we previously observed [17]. The latter can then progress to liver decompensation and the development of a state of immunosuppression known as cirrhosis‐associated immune dysfunction [7, 70]. Whether regulatory T cell expansion, myeloid‐derived suppressor cells or impaired antigen‐presenting cell function, known to be induced in cirrhosis‐associated immune dysfunction, are involved with CCl_4_ treatment prolonged beyond 12 weeks, and what influence this would have on a tumour challenge and immunotherapy at that time, remains to be determined. We did not observe a change in PD‐1 expression (marker of exhaustion) in bulk CD8 T cells throughout the CCl_4_ protocol (data not shown), suggesting exhaustion may not be the underlying reason for lack of tumour control or delayed response to immunotherapy. This may offer insight into the underlying mechanisms leading to the development of HCC in cirrhotic individuals, although an appropriately adapted model for liver cancer would more directly test such a hypothesis. As there remains a calculable risk for HCC in HCV^+^ cirrhotics, despite the success of DAA therapy, this continues to be of considerable clinical significance [71]. While the risk of HCC may decrease after DAA therapy [72, 73], some studies have observed new, and often more aggressive forms of cancer, or increased recurrence [74]. These findings further highlight the value of this model in identifying targets to restore immune function, thereby enabling effective immunotherapies for cancer. The data may provide insights into why liver cancer patients, where fibrosis is often present, may respond suboptimally to PD‐1 or CTLA‐4 immunotherapy [75, 76]. In HCC and other types of cancer, hyperresponsiveness of peripheral T cells (with no specificity for tumour antigens) has been found to strongly predict failure to immunotherapy and reduced survival [77] and associate with resistance to checkpoint inhibitor therapy in chronic hepatitis B virus infection [78]. Indeed, responses to immune checkpoint inhibitors in HCC, which often occurs in a state of cirrhosis, are often suboptimal [75, 76].

These results were complemented by a study of bulk CD8 T cell function in a HFD model of induced chronic liver damage. The 45% HFD recapitulates the liver pathology, metabolic syndrome and disease pathogenesis observed in human MASLD with advanced fibrosis, where we also observed systemic CD8 T cell hyperfunction (Figure S3). This HFD model induces little fibrosis or inflammation, decoupling it from advanced steatosis (Figure 6D,E). This is achieved by inducing glucose intolerance, subsequently resulting in increased fat mass and body weight, hence modelling obesity and metabolic dysfunction (Figure 6B,C), with apparent sex effects in observed pathologies of HFD‐fed mice (Figure S5), giving importance to the continued inclusion of sex‐stratification in ongoing and future studies of systemic CD8 T cell dysfunction. This liver insult was associated with significant increases in CD8 T cell function (Figure 6F,G), resembling that observed in the CCl_4_ model.

Given that these similar observations occurred in instances of differing pathology (i.e., HCV/CCl_4_ liver fibrosis with inflammation and HFD liver steatosis without fibrosis or inflammation), it is important to appreciate that the underlying causative mechanisms may ultimately differ. In a 60% HFD study, it was found that, after 12 weeks of diet feeding, mice had elevated CD4 Th17/Treg ratios in adipose tissue, increasing serum proinflammatory cytokines [79]. It was found that T cells are known to regulate chronic inflammation while contributing to abnormal energy metabolism [80]. While the relationship between obesity, chronic inflammation, and metabolic syndrome remains unclear, our findings implicate the phenomenon of CD8 T cell hyperfunction in liver disease‐associated metabolic syndrome. Given the common immune dysregulation in these models of chronic liver injury, clinical outcomes in cancer and immunotherapy may be compromised in steatotic liver disease.

The burden of infectious or noninfectious etiologies of chronic liver disease on human health is mounting. Over 50 million people are affected by chronic HCV infection worldwide, of which 15–30% will silently develop cirrhosis and symptomatic liver disease, predisposing to end‐stage liver disease and high mortality rates [81, 82]. It is predicted that half of HCV^+^ individuals in the USA will develop cirrhosis by 2030, often occurring before diagnosis or treatment, indicating that the relevance of research on the impact of liver damage on the host will remain significant for decades [83]. In addition, the rising burden of inflammatory fatty liver disease presents a growing challenge to global public health. The results of this study highlight a prolonged immunological scar acquired in progressive liver disease and associated with diminished antitumour responses that are refractory to immunotherapy. The use of mouse models emulating the generalized CD8 T cell dysfunction and chronic liver injury observed in humans [17] will enable mechanistic investigations and offer understanding to translate toward improving clinical outcomes of cirrhosis.

## 4 Data Limitations and Perspectives

While these results provide further insight and translational knowledge on CD8 T cell dysfunction in advanced liver disease, the associative nature of this study opens the possibility for questions on specific mechanistic relationships between liver disease pathology and blood CD8 T cell hyperfunction. Nevertheless, the results of this study, when taken together with previously reported gene expression profiling of blood CD8 T cells in human chronic HCV infection with cirrhosis [58], have established the groundwork for in‐depth mechanistic investigations, namely the possibility that chronic liver disease with advanced organ damage results in lasting epigenetic effects on bulk CD8 T cells. Comparing animal models of advanced liver disease to human liver disease builds a rationale for future mechanistic studies.

## 5 Materials and Methods

### 5.1 Animal Models of Liver Disease

To induce advanced liver fibrosis using CCl_4_, randomized 6–8 weeks old male and female mice were administered 1.0 mL/kg of CCl_4_ (Sigma‐Aldrich, USA) by intraperitoneal (i.p.) injection twice a week for ≈12 weeks, diluted in filtered food‐grade olive oil (Bertolli) to 100 µL, as an adaptation of established protocols [27, 32], following preliminary dose–response pilot studies (not shown). Liver fibrosis regression was modelled by the cessation of CCl_4_ exposure after 12 weeks, when livers reach peak fibrosis, for at least 4 weeks and up to 12 weeks.

To induce steatotic liver disease using HFD, randomized 28–30 weeks old male and female mice were placed on a 45% HFD (#D12451, Research Diets, USA) for 14 weeks, while controls were maintained on a regular chow diet (#2014, Teklad, USA). Body weight was acquired weekly, and fat and lean mass were measured relative to total body weight at week 11 using an EchoMRI‐700 (EchoMRI, USA). A glucose tolerance test was performed after 13 weeks to assess systemic glucose homeostasis and plasma insulin levels.

At endpoint, liver tissue was harvested, fixed in 10% normal buffered formalin (CCl_4_ model) or 4% paraformaldehyde (HFD model), and stored in 70% ethanol. Liver tissues were then embedded in paraffin, sectioned, and stained with Masson's trichrome (Louise Pelletier Histology Core Facility, University of Ottawa) prior to liver disease severity blinded scoring by a pathologist (Nour Histopathology Consultation Services, Ottawa, Canada). Liver damage was determined using clinically relevant pathology scoring. To assess fibrosis, the Metavir scoring system was used (F0 no fibrosis, F0‐1 minimal, F2‐3 intermediate, F3‐4 advanced fibrosis, F4 cirrhosis) [33, 34]. Liver steatosis, ballooning, and inflammation scores of 0–3 reflect states of no pathology (0), minimal (1), intermediate (2), and severe (3). Lastly, a combined non‐alcoholic fatty liver disease Activity Score, a comprehensive clinical score for MASLD‐induced liver damage also used in murine models (refs), was calculated for animals in the 45% HFD experiments [47, 48].

### 5.2 Cell Isolation and Culture

Blood samples were collected by saphenous vein draw, and red blood cells were lysed with Hybri‐Max red blood cell lysing buffer (Sigma‐Aldrich). Resulting PBMCs were cultured at 1 × 10^6^ cells/mL in 96‐well high‐binding plates (Sarstedt, USA), precoated for 1 h at 37°C with anti‐CD3 antibodies (5 µg/mL, clone: 145‐2C11, BD Biosciences, USA) and supplemented with soluble anti‐CD28 antibodies (2 µg/mL clone: 37.51, BD Biosciences). Cells were cultured for 48 h before analysis.

### 5.3 Flow Cytometry Analysis of CD8 T Cell Phenotype and Function

Six hours prior to the end of the 48 h anti‐CD3/‐CD28 cell stimulation, cells were treated with Golgi Plug (BD Biosciences), and anti‐mouse CD107a antibody was added. Next, cell viability staining was performed (Zombie viability dyes, BioLegend, USA). Cells were then labelled with cell surface receptor antibodies using the following antibodies (Clone, Fluorochrome): CD4 (GK1.5, AlexaFluor 700 or BV711), CD8α (53‐6.7, BV785), CD19 (6D5, PE‐Cy5 or BV650), CD44 (IM7, BV421), CD62L (MEL‐14, PE), PD‐1 (29F.1A12, PE‐Cy7), CD107a (1D4B, APC) from BioLegend, and NKG2D (CX5, BV711) from BD Biosciences. Cells were then fixed and permeabilized with CytoFix/CytoPerm intracellular staining kit (BD Biosciences) and labelled with anti‐mouse IFN‐γ (4S.B3, BV650 or PE‐Cy7) (BioLegend) and GrB (GB11, PE‐CF594) (BD Biosciences or BioLegend). Cells were then evaluated by flow cytometry on the LSR‐Fortessa (BD) or Aurora (Cytek) platforms, applying a fluorescence minus‐one colour compensation strategy followed by a cell subset gating strategy outlined in Figure S2. Specifically, CD8 T cell subsets were distinguished based on CD44 and CD62L expression as follows: naïve (T_N_, CD44^−^CD62L^+^), effector/effector memory (T_E/EM_, CD44^+^CD62L^−^) and central memory (T_CM_, CD44^+^CD62L^+^). Post‐acquisition flow cytometry data were analyzed using FlowJo v.10 software (FLOWJO LLC, USA).

### 5.4 Plasma Cytokine Quantitation

Mouse plasma cytokines (IFN‐γ, IL‐6, CXCL10, CCL2, CCL5, TNF) were quantified using a U‐PLEX Custom Biomarker assay (MesoScale Discovery) following the manufacturer's protocol.

### 5.5 Tumour Challenge in Chronic Liver Fibrosis

To investigate the effects of liver fibrosis on responses to tumour challenge, in some experiments, mice were injected subcutaneously (s.c.) with MC38 tumour cells (1 × 10^5^ or 1 × 10^6^ cells in 100 µL PBS). Once palpable, the ectopic tumour volume was measured every 2 days using callipers. Responses to immunotherapy experiments were performed when tumours reached ∼75 mm^3^, by i.p. injections of anti‐PD‐1 (clone RMP1‐14) and anti‐CTLA‐4 (clone 9H10) antibodies (Leinco Technologies, USA), using 200 µg of antibodies per injection in 100 µL PBS across three injections, 2 days apart.

### 5.6 Statistical Analysis

All summaries of replicate data are presented as mean ± SD unless otherwise specified. Intergroup analyses were conducted by the Student's two‐tailed *t*‐test with Welch correction where applicable (*p ≤ 0.05, **p ≤ 0.01, ***p ≤ 0.001). Multiple comparisons were tested by one‐way ANOVA with Dunnett's post‐test (*p ≤ 0.05, **p ≤ 0.01) or two‐way ANOVA with Šídák's post‐test (*p ≤ 0.05, **p ≤ 0.01), as appropriate. Microsoft Excel v.2404 (Microsoft Corporation, USA) and Prism v.10 software (Dotmatics GraphPad, USA) were used for data curation, statistical analyses, and graphical plotting. Some figure icons were obtained from BioRender (https://www.biorender.com/).

## Author Contributions

**Jood Madani**: validation, formal analysis, investigation, data curation, writing – original draft, writing – review and editing, visualization. **Jiafeng Li**: validation, formal analysis, investigation, data curation, writing – original draft, writing – review and editing, visualization. **Ma Enrica A Ching**: validation, formal analysis, investigation, data curation, writing – original draft, writing – review and editing, visualization. **Agatha Vranjkovic**: validation, formal analysis, investigation, data curation, writing – original draft, writing – review and editing. **Katrina Jorritsma**: validation, formal analysis, investigation, data curation, writing – original draft, writing – review and editing, visualization. **Mohamed S Hasim**: Formal analysis, investigation, writing – review & editing. **Manijeh Daneshmand**: methodology, formal analysis, investigation, writing – review and editing. **Angela C Cheung**: resources, writing – review and editing. **Natasha Campeau**: Writing – original draft, writing – review and editing, visualization. **David A Lawton**: Formal analysis, investigation, writing – review and editing, visualization. **Salman Bagheri**: Writing – original draft, writing – review and editing, visualization. **Erin E Mulvihill**: Methodology, resources, writing – review and editing. **Jennifer E Bruin**: Conceptualization, methodology, resources, writing – original draft, writing – review and editing, supervision, project administration, funding acquisition. **Michele Ardolino**: Methodology, resources, writing – review and editing, supervision. **Angela M Crawley**: Conceptualization, methodology, resources, writing – original draft, writing – review and editing, supervision, project administration, funding acquisition.

## Conflicts of Interest

The authors declare no conflicts of interest.

## Ethics Approval and Consent to Participate

Studies involving human samples, as well as participant enrollment and written consent, were conducted in accordance with the guidelines established by the Ottawa Health Science Network Research Board. Blood samples were collected by staff in The Ottawa Hospital Clinical Investigations Unit. Mouse studies were performed in accordance with the guidelines established by the Canadian Council on Animal Care. Hepatotoxin CCl_4_ exposure experiments were reviewed and approved by the University of Ottawa Animal Care Committee: mice were purchased from the Jackson Laboratory and housed at the University of Ottawa Animal Care and Veterinary Services facility. HFD experiments were reviewed and approved by the Carleton University Animal Care Committee: mice were bred and housed at the Carleton University vivarium.

## Supporting information




**Supporting Information file 1**: eji70026‐sup‐0001‐SuppMat.pdf

## Data Availability

The data that support the findings of this study are available from the corresponding author upon reasonable request.
