# Supplementary material for: CD8 T Cell Hyperfunction and Reduced Tumour Control in Murine Models of Advanced Liver Disease
Source: Eur J Immunol. 2025 Aug 4;55(8):e70026. doi: 10.1002/eji.70026 (PMC12322517; doi:10.1002/eji.70026)
Supplement: Supplementary file 1 — Supporting Information file 1: eji70026‐sup‐0001‐SuppMat.pdf [file EJI-55-e70026-s001.pdf]

Supplemental information

Human ethics approval and consent to participate

Studies involving human samples, as well as participant enrollment and written consent, were conducted in accordance with the guidelines established by the Ottawa Health Science Network Research Board. Blood samples were collected by staff in The Ottawa Hospital Clinical Investigations Unit.

Supplemental methods

Human CD8 T cell function in MASLD: Studies were conducted in accordance with the guidelines established by the Ottawa Health Science Network Research Ethics Board. Participants were consented prior to blood sample collection by staff at The Ottawa Hospital Clinical Investigations Unit. CD8 T cell function was assessed following previously reported approaches (58). In brief, CD8 T cells were isolated by magnetic bead positive selection from cryopreserved PBMCs and stimulated for 48h using anti-CD3/CD28 antibodies prior to flow cytometry analysis.

Liver enzyme measurements in mouse plasma: Liver AST, ALT, ALP, and TBIL were measured following previously reported methods (84), using the Beckman Coulter AU480 clinical chemistry analyzer.

Supplemental figures

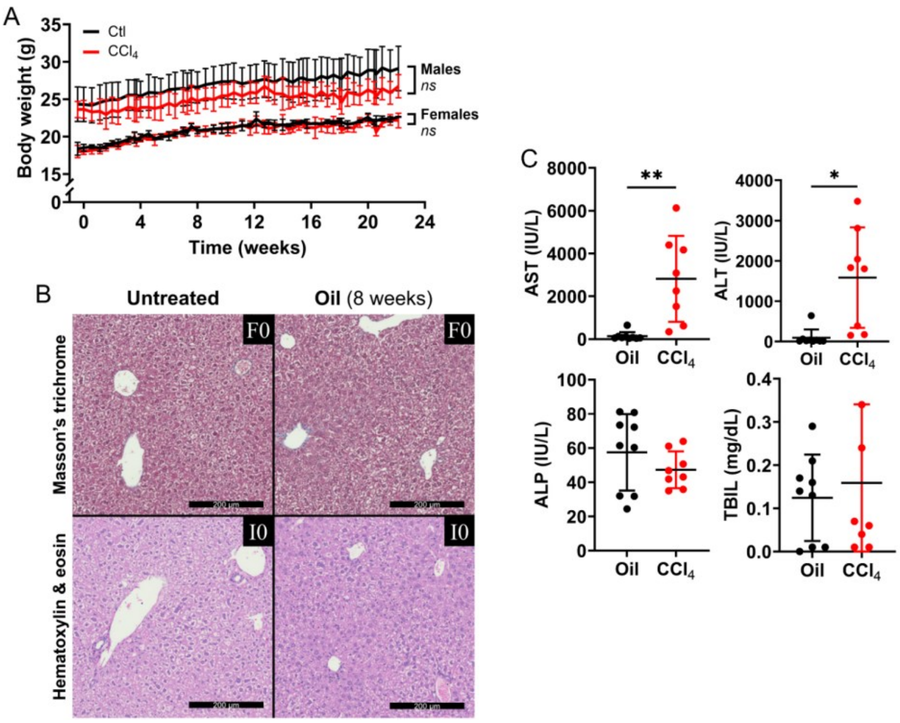

Figure S1: (A) Natural weight gain was not affected by CCl_4 exposure in both male and female mice. Comparisons by 2-way ANOVA with Šídák's post-test. **(B)** Masson's trichrome (top) and hematoxylin & eosin (bottom) staining of liver from oil vehicle-treated mice (right) did not induce visible liver damage compared to healthy untreated animals (left). Scale bars: 200 µm. **(C)** Plasma liver enzyme levels in mice with CCl_4-induced advanced liver fibrosis compared to oil controls (n=9 Oil + n=8 CCl_4). Comparisons by unpaired Student's *t*-test, $*p \leq 0.05$, $**p \leq 0.01$.

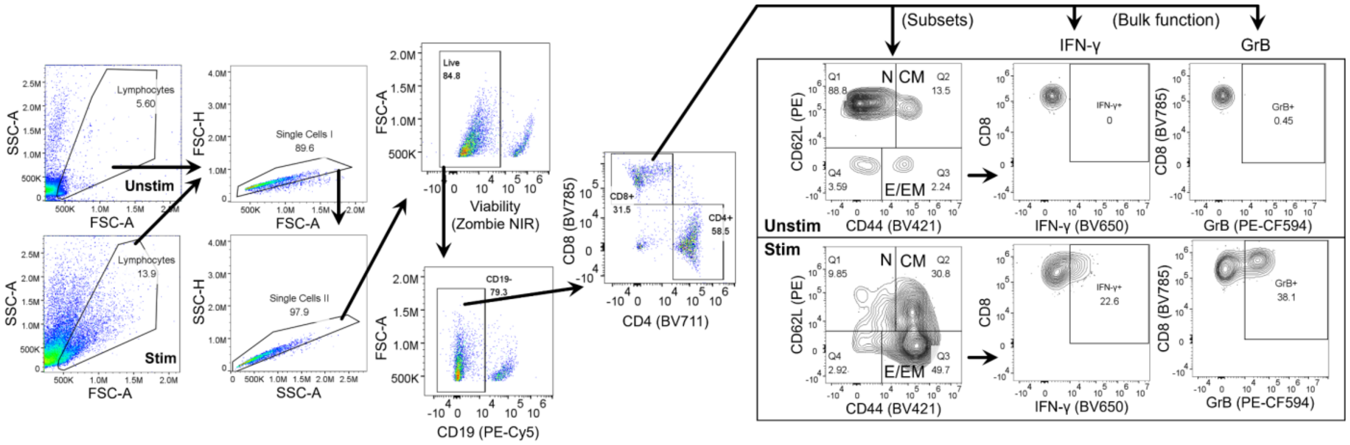

Figure S2: Gating strategy for assessing systemic CD8 T cell function in bulk cells, as well as naïve (T_N), effector/effector memory ($T_{E/EM}$), and central memory (T_{CM}) subsets.

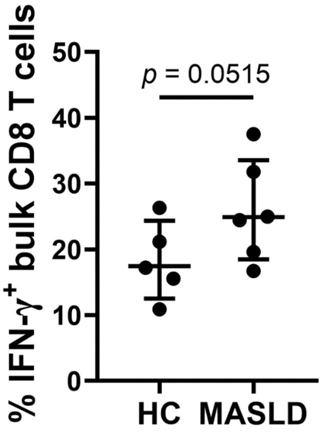

Figure S3: Preliminary findings show bulk CD8 T cell hyperfunction in human MASLD with advanced fibrosis compared to healthy controls (HC). Comparison by unpaired Student's *t*-test.

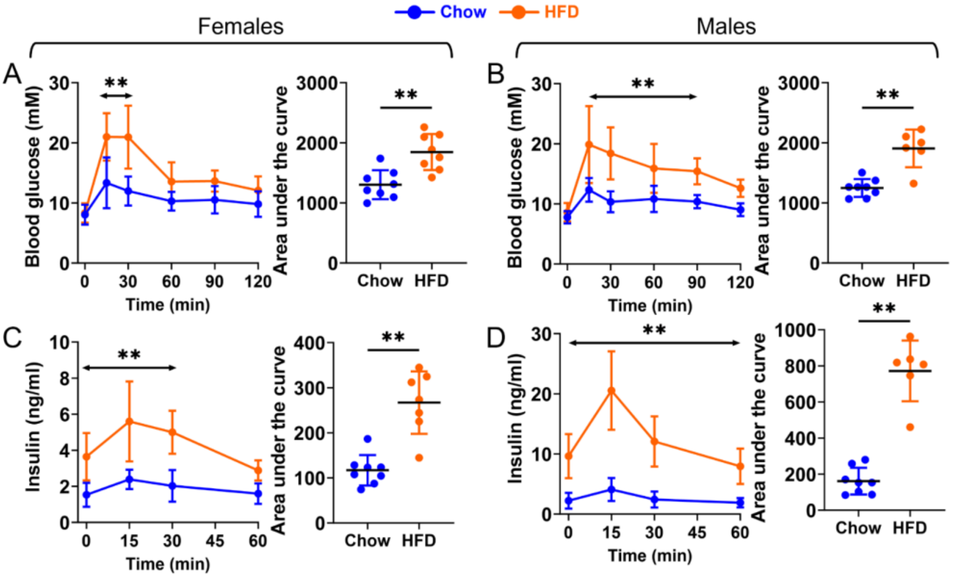

Figure S4: Top: Systemic blood glucose assessed by glucose tolerance test (GTT) after 13 weeks on HFD in **(A)** female and **(B)** male mice. **Bottom:** Corresponding plasma insulin levels during GTT in **(C)** female and **(D)** male mice. GTT responses analyzed by 2-way ANOVA with Šídák's post-test; total GTT area under the curve analyzed by unpaired Student's *t*-test; ***p*≤0.01.

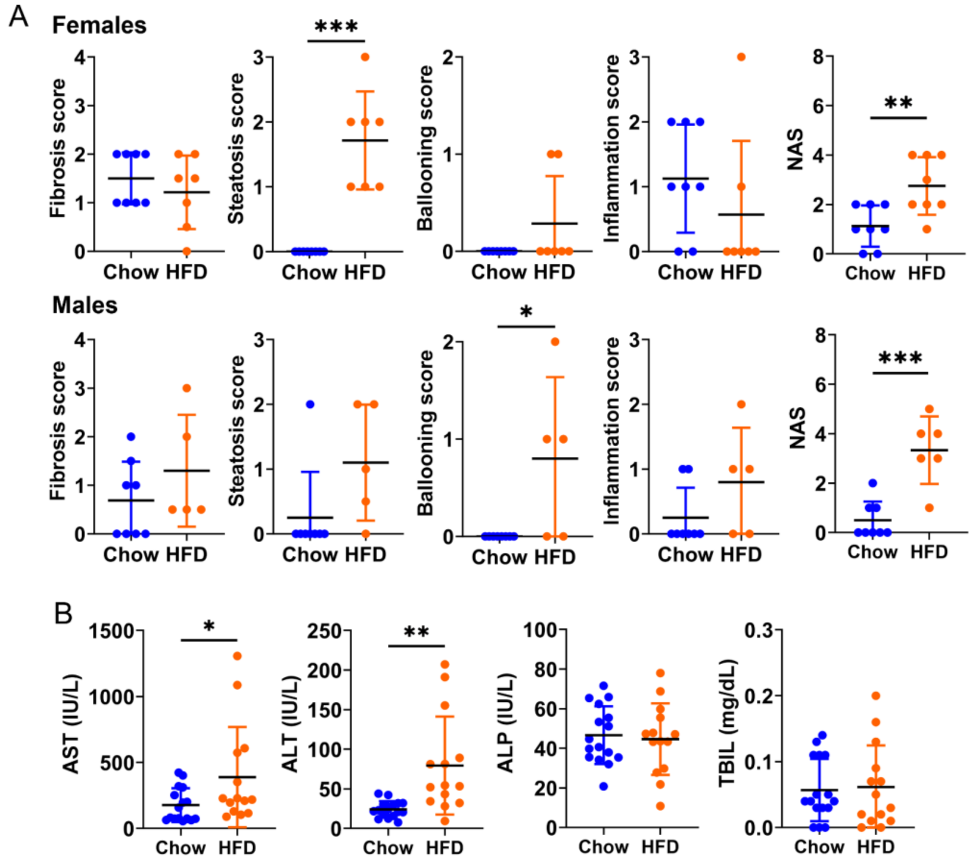

Figure S5: (A) Summarized liver disease severity scores after 14 weeks of HFD, across female (top) and male (bottom) mice. **(B)** Plasma liver enzyme levels of mice with HFD-induced steatotic liver disease compared to chow-fed controls. Comparisons by unpaired Student's *t*-test; **p*≤0.05, ***p*≤0.01, ****p*≤0.001.
